# EXAMINING RELATIONS BETWEEN OLDER ADULTS’ HEALTH, WISDOM, AND WELL-BEING DURING TIMES OF HARDSHIP

**DOI:** 10.1093/geroni/igad104.0393

**Published:** 2023-12-21

**Authors:** Monika Ardelt, Carolyn Aldwin

## Abstract

This symposium investigates the roles of health and wisdom characteristics as older adults’ resources during difficult times and whether ill health might explain lower wisdom during the later years of life. The first two presentations explored COVID-related changes in mental health. Utilizing a national sample of 1258 adults, Park found that acceptance of negative emotions, emotion regulation, and perceived social support at the beginning of the COVID pandemic (mid-April of 2020) predicted middle-aged (36-60 years, Mage=52) and older adults’ (61-88 years, Mage=70) lower distress five weeks later. Feng and colleagues analyzed well-being changes in 69 older adults (50-77 years, Mage=59) from before COVID (Time1) to the summer of 2020 (Time2) and 18 months later (Time3). Multiple logistic regressions revealed that greater wisdom and better health at Time2 and higher self-transcendence and better health at Time3 increased the likelihood of being well-adapted across time. Cogdill-Richardson and Bluck examined grief adaptation among 54 older widowed adults (Mage=81). Regression analyses showed that greater wisdom and better physical functioning predicted better grief-related mental health. Whereas good health and wisdom are consistent predictors of well-being in old age, ill health itself is a stressor. Analyzing data of 976 older adults (51-99 years, Mage = 77), Ardelt and Jeste found that the negative association between age and wisdom might be due to ill health that reduces older adults’ sense of mastery, which might lessen their motivation for wisdom development. Overall, this symposium demonstrates how health, wisdom, and well-being are interrelated, particularly during times of hardship.

